# Ameliorative Effect of Ginsenoside Rg6 in Periodontal Tissue Inflammation and Recovering Damaged Alveolar Bone

**DOI:** 10.3390/molecules29010046

**Published:** 2023-12-20

**Authors:** Won-Jin Lee, Eun-Nam Kim, Nguyen Minh Trang, Jee-Hyun Lee, Soo-Hyun Cho, Hui-Ji Choi, Gyu-Yong Song, Gil-Saeng Jeong

**Affiliations:** 1 College of Pharmacy, Chungnam National University, Daejeon 34134, Republic of Korea; wonjin2373@gmail.com (W.-J.L.); enkim@cnu.ac.kr (E.-N.K.); ngminhtrang52@gmail.com (N.M.T.); gmlwl5@naver.com (H.-J.C.); 2 AREZ Co., Ltd., Daejeon 34036, Republic of Korea; i12ulove@arez365.com (J.-H.L.); sweetylover@arez365.com (S.-H.C.)

**Keywords:** ginsenoside Rg6, *Porphyromonas gingivalis*, periodontitis, osteoblast differentiation, anti-inflammatory, antioxidant, antibacterial effects

## Abstract

Periodontal disease is a chronic disease with a high prevalence, and in order to secure natural materials to prevent oral diseases, new materials that protect periodontal tissue from inflammation are being sought. Genes were identified using real-time quantitative polymerase chain reaction (RT-qPCR), and proteins were confirmed using Western blot. Dichlorodihydrofluorescein diacetate (DCF-DA) analysis was used, and the antibacterial effects were confirmed through Minimum Inhibitory Concentration (MIC) and Minimal Bactericidal Concentration (MBC) analysis. To confirm this effect in vivo, Sprague–Dawley rats, in which periodontitis was induced using ligation or Lipopolysaccharide of *Porphyromonas gingivalis* (PG-LPS), were used. In vitro experiments using human periodontal ligament (HPDL) cells stimulated with PG-LPS showed that Ginsenoside Rg6 (G-Rg6) had anti-inflammatory, antibacterial, antioxidant, and osteoblast differentiation properties. In vivo, G-Rg6 was effective in Sprague–Dawley rats in which periodontitis was induced using ligation or PG-LPS. Therefore, Ginsenoside Rg6 shows potential effectiveness in alleviating periodontitis.

## 1. Introduction

Periodontitis is a disease in which the alveolar bone that supports the gums and teeth is destroyed by inflammation of the tooth tissue [1]. Worldwide, more than 40% suffer from periodontitis, which is characterized by the inflammation of periodontal tissue and destruction of alveolar bone and, in severe cases, causes tooth loss [2,3]. The cause of periodontitis is Gram-negative bacteria living in the oral cavity. Gram-negative bacteria include *Actinobacilius actinomycetemcomitans*, *Treponema denticola*, *Prevotella intermedia*, *Eikenella corrodens*, *Campylobacter rectus*, and *Porphyromonas gingivalis*. Among them, *Porphyromonas gingivalis* (*P. gingivalis*) is one of the most important pathogenic microorganisms associated with periodontitis [4,5]. Lipopolysaccharide (LPS) is a major component of the outer membrane of Gram-negative bacteria [6]. LPS activates Toll-like receptors (TLRs) and nucleotide-binding oligomerization domains (NODs) to induce stimulation of pro-inflammatory cytokines such as TNF-α, IL-1β, and IL-6. Then, inflammatory pathways such as the NF-κB pathway are activated, causing inflammation in the periodontal tissue [7]. In addition, reactive oxygen species, a causative agent of oxidative stress, are generated and accelerate inflammation [8]. Moreover, pro-inflammatory cytokines activate enzymes such as matrix metalloproteinases (MMPs) to produce receptor activators of nuclear factor kappa-Β ligand (RANKL) and osteoclasts and destroy alveolar bone [9]. So, the expression of alkaline phosphatase (ALP) and osteocalcin (OCN), which are indicators of bone cell activity, are important factors in alveolar bone formation and are effective in treating periodontitis by restoring bone cells lost due to periodontitis [10].

Currently, amoxicillin, penicillin, tetracycline, metronidazole, and clindamycin are non-steroidal anti-inflammatory drugs (NSAIDs) frequently used for periodontal treatment and to slow the progression of periodontitis [11]. These drugs alleviate inflammation of the gums and stop the destruction of alveolar bone. However, the side effects of these drugs are severe and side effects include gastrointestinal bleeding, high blood pressure, and liver damage [12]. In addition, cyclooxygenase-2 (COX-2) inhibitors such as celecoxib and polmacoxib are lethal to the cardiovascular and renal systems [13]. Therefore, due to the side effects of NSAIDs, a periodontal disease treatment that can replace them is needed, and natural products with a long history of use without side effects are in the spotlight [14]. Among them, as a representative example, ginsenoside Rg1 of ginseng suppressed LPS-induced inflammation in human periodontal ligament (HPDL) cells and also showed an antioxidant effect [15]. In addition, ginsenoside Rd showed inhibitory effects in terms of inflammation and bone resorption, making it valuable for controlling and preventing periodontitis [16]. However, no studies have been reported on ginsenoside Rg6 in periodontal inflammation, but G-Rg6 is known to have anti-inflammatory effects by acting broadly on systemic inflammation [17]. Thus, little investigation has been conducted on whether ginsenoside Rg6 is effective in periodontitis. Therefore, this study investigated the effects of ginsenoside Rg6 on periodontitis and alveolar bone loss in vitro and in vivo.

*Panax ginseng* C.A. Meyer belongs to the genus Panax in the Araliaceae family. Panax is the Greek word for ‘cure-all’; ginseng has the efficacy to cure all diseases [18]. Ginsenoside is the main biological component of ginseng that can treat all diseases. Ginsenoside is a triterpenoid saponin that is effective in immune regulation and has anti-inflammatory, anti-atherosclerosis, anti-hypertension, and anti-diabetes effects [19]. Ginsenoside is divided into panaxadiol, panaxatriol, β-sitosterol, and oleanolic acid [20], and ginsenoside Rg6 and ginsenoside Rh4 belong to the panaxatriol component [19]. Among them, ginsenoside Rg6 inhibits the nuclear factor kappa-Β (NF-κB) pathway and the Mitogen-Activated Protein Kinase (MAPK) pathway. In addition, bone marrow-derived macrophages express anti-inflammatory effects by expressing IL-10 [21]. Although the effects of the components of ginsenoside Rg6 (G-Rg6) are superb, there have been few studies on ginsenoside Rg6.

Therefore, in this study, we attempted to develop a new natural product material that applies to periodontitis and oral diseases, G-Rg6, and confirmed the clinical effects of G-Rg6 on various inflammatory indices in periodontitis-induced HPDL cells and investigated its effects on periodontitis and alveolar bone loss in vivo.

## 2. Results

### 2.1. Cytotoxicity Assay and Analysis of Inflammatory Factors in Inflammatory Cytokines

**Figure 1:**
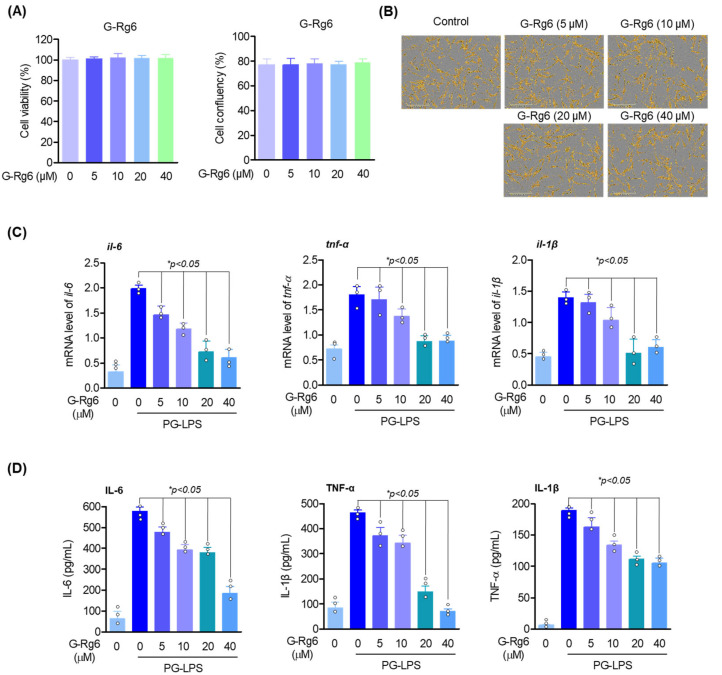
G-Rg6 showed no cytotoxicity in HPDL cells and regulated pro-inflammatory cytokines. (**A**,**B**) HPDL cells were seeded at a concentration of 5 × 10^3^ cells/mL and treated with the indicated concentrations (5–40 µM) of Rg6 for 48 h. Then, cytotoxicity experiments were investigated using MTT assay and Incucyte cell live system. (**C**) HPDL cells were seeded in a 6-well plate at a concentration of 5 × 10^5^ cells/mL, treated with the indicated concentrations of G-Rg6 for 6 h, and then stimulated for 6 h with or without PG-LPS. And then, the level of pro-inflammatory cytokine mRNA was measured by real-time PCR analysis. (**D**) The release of pro-inflammatory cytokines was analyzed by ELISA assay. * *p* < 0.05, versus the only PG-LPS-treated group.

To investigate whether G-Rg6 exhibits cytotoxic effects and anti-inflammatory activity in HPDL cells, an MTT assay was performed at concentrations of 0, 5, 10, 20, and 40 μM for 24 h. At the indicated concentrations, G-Rg6 was not cytotoxic (Figure 1A,B). In addition, to investigate the anti-inflammatory effect in HPDL cells, the effects of G-Rg6 on the expression of pro-inflammatory cytokines induced by PG-LPS were evaluated. As a result, IL-6 decreased in a concentration-dependent manner, and TNF-α and IL-1β were significantly decreased (Figure 1C). Therefore, G-Rg6 has an anti-inflammatory effect and inhibits inflammatory cytokines in HPDL. In addition, ELISA analysis was performed to evaluate the effect on the release of inflammatory cytokines directly from HPDL cells. G-Rg6 was found to be downregulated in a concentration-dependent manner (Figure 1D).

### 2.2. Exploration of Osteoblast Differentiation-Inducing Effects of G-Rg6

**Figure 2:**
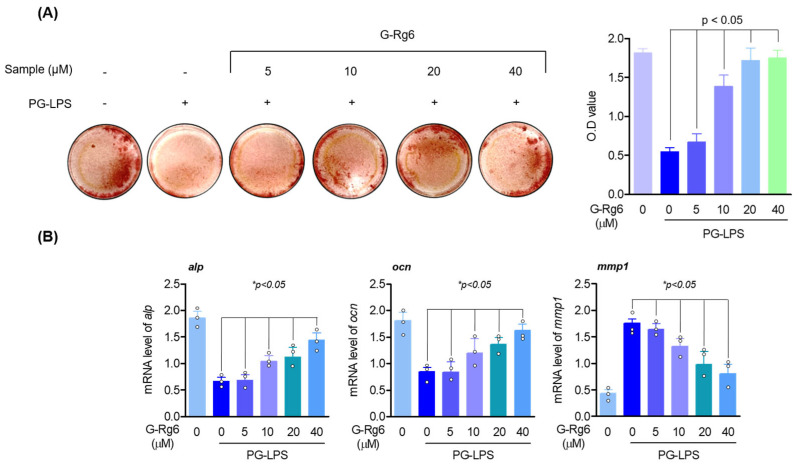
G-Rg6 upregulates osteogenic induction and osteogenic differentiation markers and downregulates MMP enzymes in HPDL cells lost due to PG-LPS. (**A**) HPDL cells were seeded in a 24-well plate at a concentration of 5 × 10^2^ cells/well. Then, the culture medium was replaced with osteoblast differentiation induction medium and incubated with G-Rg6 and PG-LPS at the indicated concentrations for 14 days. Mineralization results were measured by alizarin red staining. (**B**) The mRNA levels of ALP, OCN, and MMP-1 were measured by RT-qPCR. * *p* < 0.05, versus the only PG-LPS-treated group.

As periodontitis progresses, alveolar bone loss increases, so the recovery of ALP and OCN, which are indicators of bone cell activity, is an important treatment strategy for periodontitis treatment. Therefore, we evaluated whether G-Rg6 inhibits periodontal inflammation and alveolar bone loss in HPDL cells. First, Alizarin red S staining showed that HPDL cells, in which osteoblast differentiation was inhibited by PG-LPS, induced osteoblast differentiation by G-Rg6 (Figure 2A). In addition, to determine whether ginseng-derived natural products inhibit the gene expression of osteoblast markers such as ALP and OCN and MMP enzymes such as MMP-1 in early osteoblast differentiation, RT-qPCR was performed. The results showed that G-Rg6 increased the gene expression levels of ALP and OCN in a concentration-dependent manner and downregulated the gene expression level of MMP-1 (Figure 2B). Thus, G-Rg6 has an osteoblast differentiation-inducing effect in HPDL cells.

### 2.3. Exploring the Antioxidant Effects of G-Rg6

**Figure 3:**
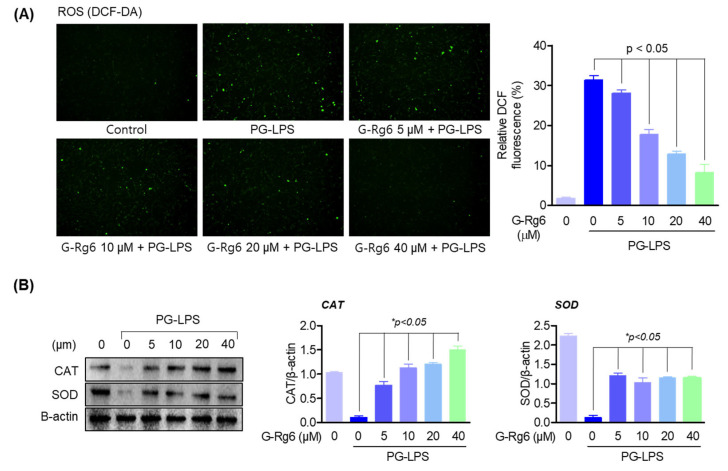
G-Rg6 regulates ROS and exhibits antioxidant effects. (**A**) HPDL cells were seeded in a 6-well plate at 5 × 10^3^ cells/mL. Natural products derived from ginseng at the indicated concentration were treated with PG-LPS and incubated for 2 h. And then ROS levels were measured by DCF-DA. (**B**) The expression of SOD and CAT proteins was measured by Western blot analysis from cells pretreated with G-Rg6 and stimulated by PG-LPS. The results were normalized to β-actin expression. * *p* < 0.05, versus the only PG-LPS-treated group.

Inhibiting the generation of reactive oxygen species (ROS) through antioxidant action is known to be an important mechanism for improving periodontitis. In HPDL cells stimulated with PG-LPS, we evaluated the effects of G-Rg6 on reactive oxygen species (ROS) scavenging and antioxidant enzyme (such as superoxide dismutase (SOD)) and catalase (CAT) production. To confirm ROS generation, it was measured by a DCF-DA assay, and G-Rg6 was downregulated in a concentration-dependent manner (Figure 3A). Additionally, G-Rg6 significantly increased the antioxidant enzymes CAT and SOD (Figure 3B). These results suggest that G-Rg6 improves periodontitis and gum health by inhibiting oxidative stress through antioxidant activity.

### 2.4. An Antibacterial Effects of P. gingivalis on G-Rg6

**Figure 4:**
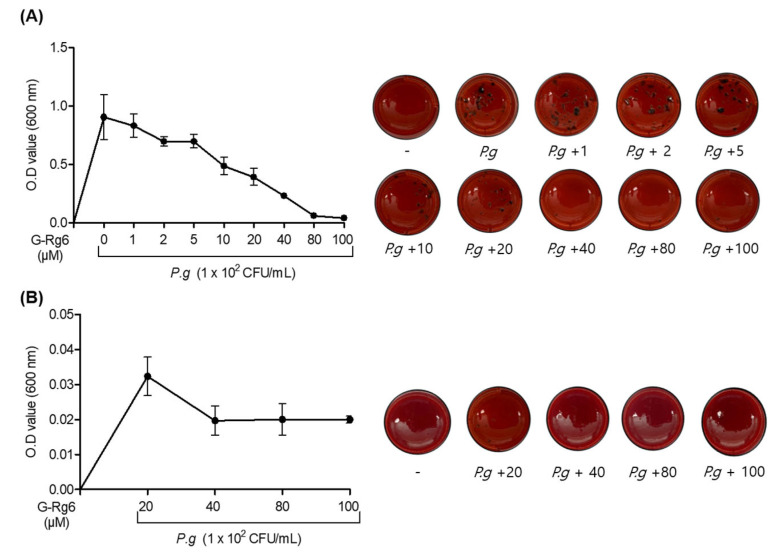
G-Rg6 demonstrates antibacterial effects and bactericidal effects against *P. gingivalis* strains. *P. gingivalis* strains were injected into TSA hemin menadione medium at a concentration of 1 × 10^2^ colony-forming units (CFU)/mL. (**A**) MIC analysis was performed by processing the indicated concentration of G-Rg6 (1, 2, 5, 10, 20, 40, 80, 100 μM). Then, for MBC analysis (**B**), the 100 μM concentration group of the ginseng-derived natural product cultured in the MIC analysis was serially diluted to create concentration groups of 20, 40, 80, and 100 μM, which were cultured and analyzed again. The antibacterial effects were measured by MIC and MBC. Absorbance wavelength was analyzed at 600 nm.

The minimal inhibition concentration (MIC) and the minimal bactericidal concentration (MBC) analyses were performed to evaluate the antibacterial effects of ginseng-derived natural products against *P. gingivalis*, a representative bacterium that causes periodontitis. As shown in Figure 4A, G-Rg6 suppressed the formation of black colonies, a morphological characteristic of *P. gingivalis* strains, from about 40 μM. Then, to evaluate whether the *P. gingivalis* strain was accurately sterilized, low and high concentrations were cultured together based on 40 and 80 μM concentrations, respectively, and the bactericidal concentration was measured. The results were identical to the minimal inhibition concentration (MBC) analysis results (Figure 4B). These results show that G-Rg6 exhibits antibacterial effects and bactericidal effects in *P. gingivalis*, a representative strain that causes periodontitis.

### 2.5. Inhibitory Effect of G-Rg6 on Periodontitis in a Ligature-Induced In Vivo Model

**Figure 5:**
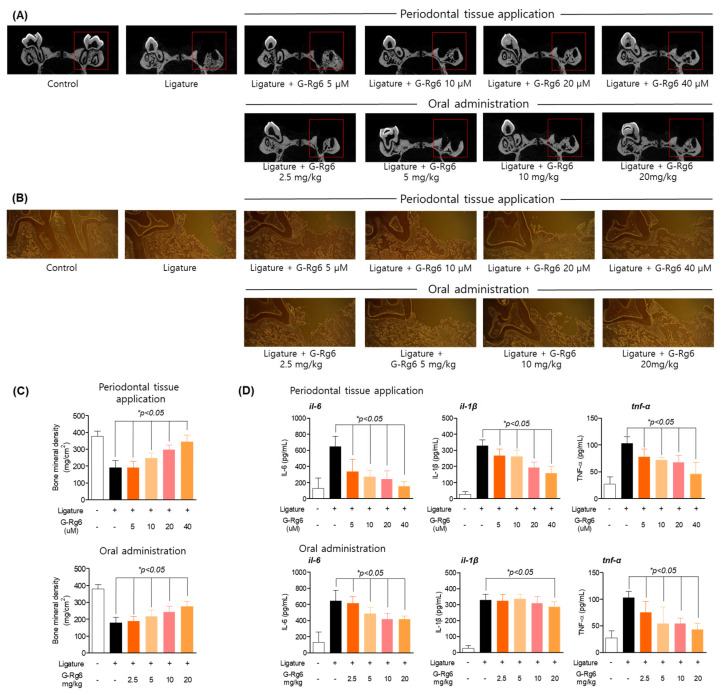
Inhibitory effects of G-Rg6 on periodontitis in a ligature-induced in vivo model. (**A**,**B**) Micro-CT (computed tomography) analysis of extraction socket (red area) and newly formed bone in the application group (upper direction) and oral administration group (bottom). Analysis tables were determined using CTAn software. (**C**) Histological analysis of the periodontium using hematoxylin and eosin (H&E) staining in the application group (upper) and oral administration group (bottom). (**D**) Confirmation of the effect of regulating inflammatory cytokines in serum in the application group (left) and oral administration group (right). * *p* < 0.05, versus the only ligature group. Scale bars denote 20 μm.

In vitro studies confirmed the effects of ginseng-derived natural products on suppressing periodontitis and inducing osteoblast differentiation. To determine the in vivo effect on periodontitis as a ligature-induced periodontitis model, the maxillary first molar was ligated with non-absorbable braid silk for 6 days to induce periodontitis. According to the results of bone mineral density (BMD), Micro-CT, and immunohistochemical staining, G-Rg6 was effective in both the oral administration group and the application group, but the application group was most effective; the socket portion of periodontal tissue lost by ligature induction was restored in a concentration-dependent manner (Figure 5A,C). In addition, the results of evaluating the efficacy of G-Rg6 against inflammatory infiltration showed that it restored the socket of periodontal tissue by ligature and downregulated inflammatory infiltration (Figure 5B). We also analyzed the degree of expression of pro-inflammatory cytokines that are representative of periodontitis (Figure 5D). The results showed that G-Rg6 significantly reduced IL-6, TNF-α, and IL-1β in both the oral administration group and the application group. According to the results, G-Rg6, a natural product derived from ginseng, effectively inhibited the ligature-induced periodontitis model. Therefore, in ligature-induced periodontitis, both the periodontal tissue application group and the oral administration group of G-Rg6 were shown to have suppressed periodontitis.

### 2.6. Inhibitory Effect of G-Rg6 on Periodontitis in a PG-LPS-Induced Periodontitis In Vivo Model

**Figure 6:**
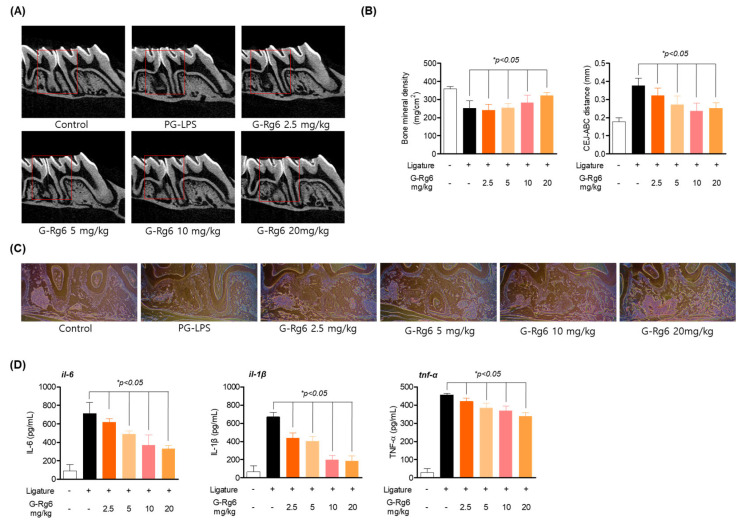
Inhibitory effects of G-Rg6 on periodontitis in PG-LPS-induced in vivo model. (**A**,**B**) Micro-CT (computed tomography) analysis of extraction socket (red area) and newly formed bone in the oral administration group and quantification of distance between cementoenamel junction (CEJ)–alveolar bone crest (ABC), and bone mineral density. Analysis tables were determined using CTAn software. (**C**) Histological analysis of the periodontium using hematoxylin and eosin (H&E) staining. (**D**) Inflammatory cytokine regulation effect in serum. * *p* < 0.05 vs. only PG-LPS-treated group. Scale bars denote 20 μm.

To corroborate the results of the ligature model, we applied PG-LPS to SD rats to induce periodontitis. According to the BMD and cementoenamel junction (CEJ)–alveolar bone crest (ABC) results, G-Rg6 appears to be significantly effective in the periodontitis model (Figure 6A,B). Micro-CT showed protected and restored periodontal root tissue and restored length of the loose periodontal crest (Figure 6B). In addition, H&E staining showed downregulated inflammatory infiltration caused by periodontitis induction and suppressed inflammatory cytokine gene levels (Figure 6C,D). Like the 2.5 results, G-Rg6 was overall effective in the PG-LPS-induced periodontitis model. This suggests that G-Rg6, a ginseng-derived natural product, is effective in alleviating periodontitis by restoring damaged periodontal tissue and alveolar bone loss in a ligature- or PG-LPS-induced periodontitis model.

## 3. Discussion

There are many drugs that stop the progression of periodontitis, but these drugs are inconvenient to use because of side effects [22]. Therefore, this study aimed to evaluate the effect of a new natural material with the ingredient ginsenoside Rg6.

Periodontitis is an inflammatory disease in which teeth are lost due to the inflammation of periodontal tissue and destruction of alveolar bone [3,23]. HPDL cells are fibroblast-like cells with characteristics of collagen production and osteoblasts and are one of the main cells of the periodontal ligament involved in the inflammatory process [24,25]. Lipopolysaccharide (LPS), a component of the cell wall of Gram-negative bacteria such as *Porphyromonas gingivalis*, causes periodontal disease [26]. Lipopolysaccharide (LPS) is a complex glycolipid composed of lipid A, a short core oligosaccharide, and an O-antigen, which can be a long polysaccharide. *Porphyromonas gingivalis* is heterogeneous and can be tetra-acylated and/or penta-acylated [27,28]. These variable regions appear to participate in inflammatory diseases and trigger various signaling pathways [29]. Thus, LPS overexpression generates excess reactive oxygen species, secretes proinflammatory cytokines, and activates osteoclasts to destroy alveolar bone [25,30,31]. Likewise, ligature also induces the necrosis of periodontal tissue and physically destroys the bond between the tooth and periodontal tissue, producing excessive reactive oxygen species and secreting pro-inflammatory cytokines, ultimately causing periodontal tissue damage and osteoclastic formation [32,33].

As a result, considering that periodontitis causes periodontal tissue destruction and alveolar bone damage by Gram-negative bacteria present in the oral cavity, important treatment strategies for periodontitis may include antibacterial effects, antioxidant effects, osteoblast differentiation, etc., and this study conducted research on the periodontitis alleviation effect of G-Rg6 using PG-LPS and ligature induction methods, which are two representative periodontitis models.

According to previous reports, various ginsenosides, including ginsenosides Rb1, Rg1, and compound K, are known to exhibit anti-inflammatory effects in various cells [34,35,36,37]. In a recent report, in mice with LPS-induced sepsis, G-Rg6 suppressed TNF-α, IL-6, and IL-1β and increased IL-10, thereby inducing the expression of miR-146a and acting as an anti-inflammatory agent in bone marrow-derived macrophages, which are known to regulate inflammatory responses [17]. However, the bioactivity of several rare ginsenosides, including G-Rg6, remains largely unknown.

Therefore, in vitro, the effects of G-Rg6 were confirmed. As a result, ginsenoside Rg6 effectively inhibited pro-inflammatory cytokines such as TNF-α, IL-1β, and IL-6 and ROS in periodontal ligament cells stimulated with PG-LPS. And ginsenoside Rg6 inhibited *P. gingivalis*, a representative strain causing periodontitis, with an inhibition concentration of 40 nM, as determined by minimal inhibition concentration (MIC) and minimal bactericidal concentration (MBC) analyses. Furthermore, as a result of checking the osteoblast differentiation ability and bone activity index, it was found that osteoblast differentiation was also promoted. Additionally, the MMP1 enzyme is an enzyme that destroys the connective tissue of the gums that occurs in periodontal disease and is said to be regulated by tissue inhibitor of metalloproteinase (TIMP) [38]. The study results showed that ginsenoside Rg6 downregulated the gene expression of *mmp-1*, which was increased by PG-LPS. Therefore, it inhibits enzymes that destroy gum connective tissue. Through this, it was confirmed that ginsenoside Rg6 is effective in anti-inflammatory, antioxidant, and antibacterial effects and osteoblast differentiation.

In the in vivo model of induced periodontitis, the effect of ginseng-derived natural products on periodontal tissue and periodontal bone loss was confirmed. The results showed that ginsenoside Rg6 was effective in bone mineral density (BMD), Micro-CT, H&E staining, and inflammatory cytokine control. Hereby, in vivo, ginsenoside Rg6 both restores periodontal tissue and periodontal bone loss. 

Therefore, in vitro results show that G-Rg6 promotes anti-inflammatory and antioxidant effects and osteoblast differentiation in HPDL cells induced by *P. gingivalis*, and also exhibits antibacterial effects. In addition, in an in vivo model induced by ligature or PG-LPS, ginsenoside Rg6 restored damaged periodontal tissue and periodontal bone loss. Currently, cyclooxygenase-2 (COX-2) inhibitors such as celecoxib and polmacoxib are fatal in cardiovascular and renal diseases, and these drugs only slow down the progression of periodontitis and are not known as a fundamental treatment method; in addition, NSAID anti-inflammatory analgesics are traditionally used to treat periodontitis, but they mainly inhibit the function of COX, which is induced by the transcriptional activity of NF-κB, and suppress only downstream signals of NF-κB [39]. However, natural products have been found to be effective in treating periodontitis by strengthening not only anti-inflammatory but also antibacterial and antioxidant defense systems [40]. In particular, ginsenoside Rg1 was shown to suppress LPS-induced inflammation in HPDLCs and also had an antioxidant effect [15]. In addition, ginsenoside Rd has been reported to be effective in controlling and preventing periodontitis by exhibiting inhibitory effects in terms of inflammation and bone resorption [16]. Therefore, ginsenosides have great potential for treating periodontitis and alveolar bone loss. According to the in vitro results of this study, G-Rg6 inhibits inflammatory cytokines and induces antibacterial and antioxidant effects and osteoblast differentiation. In addition, the in vivo experiments showed similar results to in vitro results by suppressing inflammation and differentiating osteoblasts. Therefore, it suggests that it has great potential as an adjuvant for periodontitis treatment, but additional experiments are needed to determine what molecular mechanism G-Rg6 acts through.

## 4. Conclusions

This study demonstrates the anti-periodontitis effect of G-Rg6 and shows new biological activity of G-Rg6 in periodontitis. The study results showed that G-Rg6 not only regulated the expression of inflammatory cytokines and antioxidant and antibacterial mediators in HPDL cells stimulated with PG-LPS but also increased the induction of osteogenesis. In addition, in an animal model of periodontitis induced by PG-LPS and ligature, it showed an inhibitory effect on periodontitis by restoring lost periodontal tissue and controlling inflammation. Therefore, we propose the potential of G-Rg6 as a new natural product-derived periodontitis control agent.

## 5. Materials and Methods

### 5.1. Chemicals and Reagents

The ginsenoside Rg6 (G-Rg6) compound was provided by AREZ Co., Ltd. (Daejeon, Republic of Korea). Minimum Essential Medium-Alpha (α-MEM), fetal bovine serum (FBS), penicillin/streptomycin, and trypsin-ethylene diamine tetra acetic acid (EDTA) were purchased from Gibco (Grand Island, NY, USA). MTT (3-[4,5-dimethylthiazol-2-yl]-2,5-diphenyl tetrazolium bromide) was purchased from Amresco Inc. (Cleveland, OH, USA). Lipopolysaccharide isolated from *P. gingivalis* (PG-LPS) was obtained from InvivoGen (San Diego, CA, USA). Enzyme-Linked Immunosorbent Assay (ELISA) kits were obtained from R & D system (Minneapolis, MN, USA). TNF-α, IL-6, and IL-1β were also obtained from R & D system. For Western blot analysis, a Hybond ECL PVDF was purchased from Amersham Pharmacia Biotech Inc. (Piscataway, NJ, USA). To detect Western blotting, a Western blotting detection system was purchased from Advansta Inc. (Santa Clara, CA, USA). For osteoblast staining, Alizarin Red S was purchased from Sigma–Aldrich (St. Louis, MO, USA). To detect reactive oxygen species (ROS), 2′, 7′-Dichlorofluorescin diacetate (DCF-DA) was purchased from Sigma–Aldrich (St. Louis, MO, USA).

### 5.2. Cell Culture

The HPDL (human periodontal ligament) cells were provided by Professor Jae-Young Kim at Kyungpook National University, College of Dentistry. The protocols for the isolation and culture of HPDL (human periodontal ligament) cells were reviewed and approved by the Institutional Review Board of Kyungpook National University (Daegu, Republic of Korea) (KNU 2017-78). HPDL cells were cultured in α-MEM supplemented with 10% (*v*/*v*) fetal bovine serum (FBS) and 1% penicillin/streptomycin (Gibco BRL, Grand Island, NY, USA), and the cells were maintained at 37 °C. 

### 5.3. Plant Materials

The ginseng berries used in this study were purchased by CK Pharm CO., LTD. (Chungcheong bukdo, Republic of Korea, 2022). It was identified as a ginseng berry by Soon-Hyang Nam, production general manager of CK Pharm CO., LTD. The specimen of ginseng berry from AREZ Co. Ltd. was stored in the laboratory (Voucher No: GB220523KR).

### 5.4. Preparation of G-Rg6

A total of 5.0 kg of ginseng berry, in 60 L of 50% ethyl alcohol, was extracted three times under 75–85 °C for 8 h, 5 h, and 5 h. The extracts were concentrated at 50–60 °C and 50 mmHg and 60 brix concentrate (3.08 kg) was obtained. This concentrate was isolated using HP-20 resin, a synthetic absorbent, to obtain 255 g of ginsenoside Rg (purity > 98%, Code No: ARRE_20220901).

Ten grams of ginsenoside Rg (Code No: ARRE_20220901) in 20 mL of distilled water were steamed for 7 h under 121 °C and 0.13 Mpa. The steamed crude saponin was adsorbed on an RP-C18 column (Cosmosil 75C18) and chromatographed on an RP-C18 column (Biotage^®^ Sfär C18 D Duo 100 Å 30 μm 120 g, X2) by MPLC eluting with H_2_O and MeOH (10–45% Acetonitrile, 10 L) to obtain pure rare ginsenoside Rg6 (2.22 g, purity > 96%, Code No: ARRG6_221107). The separation was achieved by gradient elution as follows: 0–50 min (40–50% B), 50–110 min (50–55% B), 110–180 min (55% B) A (H_2_O) and B (MeOH). The flow rate of the mobile phase was 50 mL/min. The wavelength was set at 205 nm, 200–220 nm range. The purity of ginsenoside Rg6 was determined to be 96% by analytical HPLC. HPLC analysis was performed using a Shimadzu Nexera series with a UV detector (205 nm) and an ACE 5-C18 column (250 × 4.6 mm). The separation was achieved by gradient elution as follows: 0–25 min (45% B), 25–30 min (45–80% B), 30–45 min (80–45% B) A (H_2_O) and B (Acetonitrile). The solvent flow rate was held constant at 1 mL/min, and the sample injection volume was 20 μL.

Ginsenoside Rg6: (3β,6α,12β)-3,12-Dihydroxydammara-20,24-dien-6-yl-2-O-(6-deoxy-β-L-mannopyranosyl)-β-D-glucopyranoside, white powder, C_42_H_70_O_12_, HR-FAB-MS: *m*/*z* = 789.4767 [M + Na]^+^ (Figure S1).

^1^H NMR (400 MHz, Pyridine-*d*_5_) δ 6.50 (br s, 1H), 5.33 (d, *J* = 8.1 Hz, 1H), 5.29 (d, *J* = 6.8 Hz, 1H), 5.13 (br s, 1H), 4.97 (m, 1H), 4.97 (m, 1H), 4.80 (br s, 1H), 4.72 (m, 1H), 4.72 (m, 1H), 4.54 (m, 1H), 4.37 (m, 4H), 4.23 (t, 1H), 3.95 (m, 1H), 3.95 (m, 1H), 3.50 (m, 1H), 2.95 (m, 1H), 2.79 (m, 2H), 2.13 (s, 3H), 2.05 (m, 4H), 1.92 (m, 4H), 1.80 (s, 3H), 1.68 (s, 3H), 1.65 (m, 1H), 1.61 (s, 3H), 1.47 (m, 3H), 1.42 (m, 3H), 1.39 (s, 3H), 1.28 (s, 3H), 1.23 (m, 1H), 1.01 (s, 3H), 1.00 (s, 3H), 0.99 (m, 1H), ^13^C NMR (100 MHz, Pyridine-*d*_5_) δ 155.9, 131.6, 125.7, 108.5, 102.3, 102.2, 79.8, 78.9, 78.8, 78.7, 74.7, 74.6, 73.0, 73.0, 72.8, 72.7, 69.8, 63.5, 61.2, 52.5, 51.6, 50.6, 48.6, 46.6, 41.7, 40.4, 40.1, 39.9, 34.1, 33.1, 33.0, 32.6, 31.1, 28.1, 27.4, 26.1, 19.1, 18.1, 18.0, 17.6, 17.5, 17.3 (Figure S2).

### 5.5. Cell Viability and Live Cell Analysis

HPDL cells (5 × 10^3^ cells/mL) were seeded in a 96-well plate for 24 h (37 °C, 5% CO_2_). Then, HPDL cells were treated with G-Rg6 for 48 h at concentrations of 5, 10, 20, and 40 μM. Then, after, 5 mg/mL MTT (3-[4,5-dimethylthiazol-2-yl]-2,5-diphenyl tetrazolium bromide) was added to 100 μL of cell suspension, followed by incubation for 4 h. The medium was removed and 200 μL dimethyl sulfoxide (DMSO) was added. The absorbance of dissolved formazan crystals was measured by a microplate reader (Tecan Trading AG) (Männedorf, Switzerland) at 540 nm. For coefficient assays, cells were counted with the Incucyte^®^ Live-Cell analysis system (Göttingen, Germany).

### 5.6. ELISA Assay

HPDL cells were cultured at a density of 5 × 10^4^ cells/well in 96-well microplates and then treated with Rg6 at 5, 10, 20, and 40 μM for 6 h. Then, after treatment with PG-LPS for 18 h, the supernatant was taken according to the manufacturer’s instructions, and the secreted amounts of inflammatory cytokines, IL-6, IL-1b, and TNF-α were measured with an ELISA kit (R&D system, Minneapolis, MN, USA).

### 5.7. Alizarin Red Staining

HPDL cells were cultured in a 24-well culture plate at 5 × 10^2^ cells/well, and the culture medium was removed after 24 h. Osteoblast differentiation was induced by replacing the medium with α-MEM containing 10% fetal bovine serum, 100 U/mL penicillin, 100 ug/mL streptomycin, 50 µg/mL ascorbic acid, and 10 mM β-glycerophosphate. They were treated with the indicated concentrations (μg/mL) of G-Rg6 and PG-LPS (1 μg/mL) while inducing and cultured for 14 days at 37 °C under 5% CO_2_ culture conditions. Then, they were fixed with 4% polyformaldehyde for 30 min and stained with 0.1% Alizarin Red S (Sigma–Aldrich, St. Louis, MO, USA) for 1 h followed by washing with deionized water for 30 min at room temperature. Mineralized nodule formation was confirmed through a microscope, dissolved with 400 µL of 10% cetylpyridinium chloride, and absorbance at 570 nm was measured.

### 5.8. Determination of Intracellular Reactive Oxygen Species (ROS)

The reactive oxygen species (ROS) generation was assessed with DCF-DA (sigma D6883, St Louis, MO, USA). HPDL cells were cultured in a 6-well plate at a concentration of 5 × 10^3^ cells/mL for 24 h (37 °C, 5% CO_2_). The indicated concentrations of G-Rg6 (5, 10, 20, 40 μM) and PG-LPS (1 μg/mL) were treated and incubated for 2 h. After, cells were treated with medium supplemented with 10 μM DCF-DA and incubated for 30 min. The cells were washed with PBS and treated with 1% trypsin–EDTA solution to harvest the cells. Then, they were washed again with PBS, treated, and cultured for 30 min. After that, changes in ROS were measured using the Incucyte^®^ Live-Cell analysis system.

### 5.9. Western Blot Analysis

HPDL cells were cultured in 6-well plates at 5 × 10^5^ cells/mL for 24 h (37 °C, 5% CO_2_). The samples were treated for 6 h and then stimulated with PG-LPS for 12 h. After that, HPDL cells were washed with PBS followed by lysis by RIPA (Radio-Immunoprecipitation Assay) buffer for 30 min. And lysates were centrifuged at 12,000 rpm for 15 min. To determine the protein concentration, a Bradford assay (Sigma Alrich, St. Louis, MO, USA) was performed. Then, an equal amount of protein (20 μg) was separated by 12% SDS-PAGE and transferred to PVDF membranes. Then, the membranes were blocked at room temperature for 1 h with 5% (*w*/*v*) non-fat dried milk dissolved in TBST buffer (10 mM Tris (pH 8.0) and 150 mM NaCl). After blocking, the membranes were rinsed with TBST buffer for 1 h and incubated with primary monoclonal antibodies of Superoxide Dismutase (SOD) and Catalase (CAT) overnight at 4 °C for 24 h. The membranes incubated with the primary antibody were washed with TBST buffer and incubated with mouse or rabbit secondary antibody (Santa Cruz, CA, USA). Proteins activated by binding to the antibody were measured using an ECL Western blotting detection system. The primary antibodies against CAT and SOD were acquired from BD Biosciences (San Jose, CA, USA). B-actin was purchased from Santa Cruz Biotechnology, Inc. (Santa Cruz, CA, USA)

### 5.10. RT-qPCR Analysis

HPDL cells were cultured in 6-well plates at 5 × 10^5^ cells/mL for 24 h (37 °C, 5% CO_2_). As shown in Table 1, the samples were treated for 6 h and then stimulated with PG-LPS for 12 h. Total RNA from HPDL cells treated with G-Rg6 was extracted using a TRIzol/chloroform reagent (Bioneer, Daejoner, Republic of Korea). To measure the expression level of each gene, total RNA was extracted from cell lysates with TRIZOL reagent and quantified using NanoDrop (Thermo science, Waltham, MA, USA); then, TOPscript™ RT DryMIX (dT18 plus) was used to measure the expression level of each cDNA synthesized. Afterward, PCR reactions were performed utilizing a LightCycler 480 (Roche, Basel, Switzerland). Real-time PCR was used to measure mRNA for each target, and cycle threshold (Ct) values of target genes were normalized to GAPDH using GAPDH as a housekeeping gene. Primers for each gene were obtained from Biomedic Co (Bucheon, Republic of Korea), the experiment was repeated three times independently, and the primer sequences are shown in Table 1.

### 5.11. Determination of the Minimal Inhibition Concentration (MIC) and the Minimal Bactericidal Concentration (MBC)

The strain of *P. gingivalis* (KCTC 5352) used in the study was obtained from the Biological Resources Center of the Jeonbuk Branch of the Korea Research Institute of Bioscience and Biotechnology. First, the *P. gingivalis* strain was injected into Tryptic Soy Agar (TSA) hemin menadione medium at a concentration of 1 × 10^2^ colony-forming units (CFU)/mL. Then, they were treated with indicated concentrations of G-Rg6 (1, 2, 5, 10, 20, 40, 80, 100 μM) and cultured at 37 °C under anaerobic conditions. After the end of incubation, absorbance was analyzed at 600 nm. After MIC analysis, for MBC analysis, the G-Rg6 100 μM concentration group cultured by MIC analysis was serially diluted to create concentration groups of 20, 40, and 80 μM. Then, absorbance was analyzed at 600 nm.

### 5.12. Animals

In this study, 7-week-old male Sprague–Dawley rats were purchased from Samtako Inc. (Osan, Republic of Korea) to establish a periodontitis in vivo model, and the study was conducted at the Joint Animal Experiment Center of Chungnam National University (Daejeon, Republic of Korea). All animal experiments in this study were approved by the Animal Experiment Ethics Committee of Chungnam National University, and the approval number is 202212-CNU-259.

### 5.13. Ligature-Induced Periodontitis Model

Male 7-week-old Sprague–Dawley rats were ligated with non-absorbable braided silk for 5 days around the second molars. After 5 days, the ligature was removed along with the maxillary first molars in the application group, and G-Rg6 (5, 10, 20, 40 μM) was applied for 9 days. Additionally, in the case of the oral administration group, G-Rg6 (2.5, 5, 10, 20 mg/kg) was administered orally every day for 9 days. After completion of the experiment, the maxilla was obtained from the sacrificed rat and washed with phosphate-buffered saline. Afterward, the palate flesh was removed and washed with a phosphate-buffered saline solution. Afterward, imaging of the maxilla was performed using 2D/3D micro-CT.

### 5.14. PG-LPS-Induced Periodontitis Model

Sprague–Dawley rats were injected with PG-LPS between the maxillary first and second molars to induce periodontitis for 6 days and then G-Rg6 at doses of 2.5, 5, 10, and 20 mg/kg was administered for 14 days. It was administered orally. Each experimental group was divided into six groups as follows. (1) CON (PBS injection); (2) PG-LPS (6 days induced and P.O PBS injection for 14 days); (3) PG-LPS (6 days induced) + sample (injected G-Rg6 2.5 mg/kg for 14 days); (4) PG-LPS (6 days induced) + sample (P.O, G-Rg6 5 mg/kg for 14 days); and (5) PG-LPS (6 days induced) + sample (P.O, G-Rg6 10 mg/kg for 14 days); and (6) PG-LPS (6 days induced) + sample (P.O, G-Rg6 20 mg/kg for 14 days). After completion of the experiment, the experimental animals were sacrificed and the maxillary first molars were removed, and the experimental method was the same as Section 5.13.

### 5.15. Micro-CT Imaging and Analysis

After completion of the experiment, serum and maxillary skulls were obtained from sacrificed rats for Micro-CT (Quantum FX micro-CT, Perkin Elmer, Waltham, MA, USA) imaging. Micro-CT’s analysis conditions were tube voltage (100 kV), tube current (200 µA), imaging time (180 s), scanning FOV (field of view, 5 mm), and pixel size (10 μM). To measure bone mineral density (BMD), the direction was changed to a coronal section, the image was loaded into CTAn, and the scanned image was calculated as bone density (BMD) based on the above threshold. Additionally, a region of interest (ROI) was set using the interpolation method of the alveolar bone excluding the area from the cement–enamel junction to the tooth root, and the data for each group were expressed as mean ± SD.

### 5.16. Histological Staining

Hematoxylin and eosin (H&E) staining was performed to confirm periodontal invasion of the periodontal tissue of the extracted maxillary skull. The entire extracted maxillary skull was fixed in 10% formalin and then embedded in paraffin. Afterward, it was cut into 5 μm sections and fixed on a slide, and the tissue fixed on the slide was stained with H&E. The tissue fixed on the slide was then observed for the degree of periodontal tissue invasion using a fluorescence Olympus IX microscope 71-F3 2PH (Tokyo, Japan).

### 5.17. Statistical Analysis

Each experiment in this study was performed at least in triplicate, and statistical analysis of all data was performed using GraphPad Prism 5 software (San Diego, CA, USA). Additionally, *p* < 0.05 for the mean value and standard deviation (SD) was considered to indicate statistical significance.

## Figures and Tables

**Table 1:** Primer sequences.

Target Gene	Sequence (5′→3′)	
*IL-6*	Forward	AGTGAGGAACAAGCCAGAGC
	Reverse	GTCAGGGGTGGTTATTGCAT
*IL-1Β*	Forward	AACCTCTTCGAGGCACAAGG
	Reverse	GTCCTGGAAGGAGCACTTCAT
*TNF-A*	Forward	GCCTCTTCTCCTTCCTGATCGT
	Reverse	TGAGGGTTTGCTACAACATGGG
*ALP*	Forward	TGCAGTACGAGCTGAACAGG
	Reverse	GTCAATTCTGCCTCCTTCCA
*MMP-1*	Forward	ATGAAGCAGCCCAGATGTGGAG
	Reverse	TGGTCCACATCTGCTCTTGGCA
*OCN*	Forward	CGCTACCTGTATCAATGGCTGG
	Reverse	CTCCTGAAAGCCGATGTGGTCA
*GAPDH*	Forward	TGTTCGTCATGGGTGTGAAC
	Reverse	GTCTTCTGGGTGGCAGTGAT

## Data Availability

All data generated or analyzed during this study are included in this published article.

## Supplementary Materials

The following supporting information can be downloaded at: https://www.mdpi.com/article/10.3390/molecules29010046/s1, Figure S1: Mass spectrometry of G-Rg6; Figure S2: Nuclear Magnetic Resonance (NMR) spectrum of G-Rg6.

## References

[1] Pihlstrom B.L., Michalowicz B.S., Johnson N.W. (2005). Periodontal diseases. Lancet.

[2] Chen M.X., Zhong Y.J., Dong Q.Q., Wong H.M., Wen Y.F. (2021). Global, regional, and national burden of severe periodontitis, 1990-2019: An analysis of the Global Burden of Disease Study 2019. J. Clin. Periodontol..

[3] Williams R.C. (1990). Periodontal disease. N. Engl. J. Med..

[4] Lovegrove J.M. (2004). Dental plaque revisited: Bacteria associated with periodontal disease. J. N. Z. Soc. Periodontol..

[5] Mysak J., Podzimek S., Sommerova P., Lyuya-Mi Y., Bartova J., Janatova T., Prochazkova J., Duskova J. (2014). *Porphyromonas gingivalis*: Major periodontopathic pathogen overview. J. Immunol. Res..

[6] Chiang C.Y., Kyritsis G., Graves D.T., Amar S. (1999). Interleukin-1 and tumor necrosis factor activities partially account for calvarial bone resorption induced by local injection of lipopolysaccharide. Infect. Immun..

[7] Graves D.T., Oates T., Garlet G.P. (2011). Review of osteoimmunology and the host response in endodontic and periodontal lesions. J. Oral Microbiol..

[8] Chen Y., Zhang Q., Qin X., Li J., Zhao Y., Xia Y. (2022). Superparamagnetic Iron Oxide Nanoparticles Protect Human Gingival Fibroblasts from *Porphyromonas gingivalis* Invasion and Inflammatory Stimulation. Int. J. Nanomed..

[9] Zhang Z., Song J., Kwon S.H., Wang Z., Park S.G., Piao X., Ryu J.H., Kim N., Kim O.S., Kim S.H. (2023). Pirfenidone Inhibits Alveolar Bone Loss in Ligature-Induced Periodontitis by Suppressing the NF-κB Signaling Pathway in Mice. Int. J. Mol. Sci..

[10] Kızıldağa A., Alpanb A.L., Özdedec M., Aydınd T., Özmene Ö. (2023). Therapeutic effects of diosgenin on alveolar bone loss and apoptosis in diabetic rats with experimental periodontitis. Iran. J. Basic Med. Sci..

[11] Lee B.A., Lee H.S., Jung Y.S., Kim S.W., Lee Y.W., Chang S.H., Chung H.J., Kim O.S., Kim Y.J. (2013). The effects of a novel botanical agent on lipopolysaccharide-induced alveolar bone loss in rats. J. Periodontol..

[12] Herrera D., Sanz M., Jepsen S., Needleman I., Roldán S. (2002). A systematic review on the effect of systemic antimicrobials as an adjunct to scaling and root planing in periodontitis patients. J. Clin. Periodontol..

[13] Kirschneck C., Wolf F., Cieplik F., Blanck-Lubarsch M., Proff P., Schröder A. (2020). Impact of NSAID etoricoxib on side effects of orthodontic tooth movement. Ann. Anat..

[14] Kumar R., Mirza M.A., Naseef P.P., Kuruniyan M.S., Zakir F., Aggarwal G. (2022). Exploring the Potential of Natural Product-Based Nanomedicine for Maintaining Oral Health. Molecules.

[15] Chu K., Zhang Z., Chu Y., Xu Y., Yang W., Guo L. (2023). Ginsenoside Rg1 alleviates lipopolysaccharide-induced pyroptosis in human periodontal ligament cells via inhibiting Drp1-mediated mitochondrial fission. Arch. Oral Biol..

[16] Zhou S., Ji Y., Yao H., Guo H., Zhang Z., Wang Z., Du M. (2022). Application of Ginsenoside Rd in Periodontitis with Inhibitory Effects on Pathogenicity, Inflammation, and Bone Resorption. Front. Cell Infect. Microbiol..

[17] Paik S., Choe J.H., Choi G.E., Kim J.E., Kim J.M., Song G.Y., Jo E.K. (2019). Rg6, a rare ginsenoside, inhibits systemic inflammation through the induction of interleukin-10 and microRNA-146a. Sci. Rep..

[18] Rokot N.T., Kairupan T.S., Cheng K.C., Runtuwene J., Kapantow N.H., Amitani M., Morinaga A., Amitani H., Asakawa A., Inui A. (2016). A Role of Ginseng and Its Constituents in the Treatment of Central Nervous System Disorders. Evid. Based Complement. Alternat. Med..

[19] Christensen L.P. (2009). Ginsenosides chemistry, biosynthesis, analysis, and potential health effects. Adv. Food Nutr. Res..

[20] Wu W., Sun L., Zhang Z., Guo Y., Liu S. (2015). Profiling and multivariate statistical analysis of *Panax ginseng* based on ultra-high-performance liquid chromatography coupled with quadrupole-time-of-flight mass spectrometry. J. Pharm. Biomed. Anal..

[21] Shin B.K., Kwon S.W., Park J.H. (2015). Chemical diversity of ginseng saponins from *Panax ginseng*. J. Ginseng Res..

[22] Im D.S. (2020). Pro-Resolving Effect of Ginsenosides as an Anti-Inflammatory Mechanism of *Panax ginseng*. Biomolecules.

[23] Heta S., Robo I. (2018). The Side Effects of the Most Commonly Used Group of Antibiotics in Periodontal Treatments. Med. Sci..

[24] Luo Y., Yang B., Dong W., Yu W., Jia M., Wang J. (2023). DNA damage-inducible transcript 3 deficiency promotes bone resorption in murine periodontitis models. J. Periodontal Res..

[25] Socransky S.S., Haffajee A.D., Cugini M.A., Smith C., Kent R.L. (1998). Microbial complexes in subgingival plaque. J. Clin. Periodontol..

[26] Wu C., Xia L., Zhang B., Bai Z., Yuan L., Xu D. (2023). Astragaloside reduces toxic effect of periodontal ligament fibroblasts induced by lipopolysaccharide. Arch. Biochem. Biophys..

[27] Zhang Z., Zhang Y., Cai Y., Li D., He J., Feng Z., Xu Q. (2023). NAT10 regulates the LPS-induced inflammatory response via the NOX2-ROS-NF-κB pathway in macrophages. Biochim. Biophys. Acta Mol. Cell Res..

[28] Ogawa T., Asai Y., Makimura Y., Tamai R. (2007). Chemical structure and immunobiological activity of *Porphyromonas gingivalis* lipid A. Front. Biosci..

[29] Fernández A., Herrera D., Hoare A., Hernández M., Torres V.A. (2023). Lipopolysaccharides from Porphyromonas endodontalis and *Porphyromonas gingivalis* promote angiogenesis via Toll-like-receptors 2 and 4 pathways in vitro. Int. Endod. J..

[30] Olsen I., Singhrao S.K. (2018). Importance of heterogeneity in *Porhyromonas gingivalis* lipopolysaccharide lipid A in tissue specific inflammatory signalling. J. Oral Microbiol..

[31] Pan W., Wang Q., Chen Q. (2019). The cytokine network involved in the host immune response to periodontitis. Int. J. Oral Sci..

[32] Kook K.E., Kim C., Kang W., Hwang J.K. (2018). Inhibitory Effect of Standardized *Curcuma xanthorrhiza* Supercritical Extract on LPS-Induced Periodontitis in Rats. J. Microbiol. Biotechnol..

[33] Wang Z., Luo W., Zhang G., Li H., Zhou F., Wang D., Feng X., Xiong Y., Wu Y. (2023). *FoxO1* knockdown inhibits RANKL-induced osteoclastogenesis by blocking NLRP3 inflammasome activation. Oral Dis..

[34] Jönsson D., Nebel D., Bratthall G., Nilsson B.O. (2011). The human periodontal ligament cell: A fibroblast-like cell acting as an immune cell. J. Periodontal Res..

[35] Park E.K., Shin Y.W., Lee H.U., Kim S.S., Lee Y.C., Lee B.Y., Kim D.H. (2005). Inhibitory effect of ginsenoside Rb1 and compound K on NO and prostaglandin E2 biosyntheses of RAW264.7 cells induced by lipopolysaccharide. Biol. Pharm. Bull..

[36] Du J., Cheng B., Zhu X., Ling C. (2011). Ginsenoside Rg1, a novel glucocorticoid receptor agonist of plant origin, maintains glucocorticoid efficacy with reduced side effects. J. Immunol..

[37] Park E.K., Choo M.K., Han M.J., Kim D.H. (2004). Ginsenoside Rh1 possesses antiallergic and anti-inflammatory activities. Int. Arch. Allergy Immunol..

[38] Balli U., Cetinkaya B.O., Keles G.C., Keles Z.P., Guler S., Sogut M.U., Erisgin Z. (2016). Assessment of MMP-1, MMP-8 and TIMP-2 in experimental periodontitis treated with kaempferol. J. Periodontal Implant. Sci..

[39] Jang W.Y., Hwang J.Y., Cho J.Y. (2023). Ginsenosides from Panax ginseng as Key Modulators of NF-κB Signaling Are Powerful Anti-Inflammatory and Anticancer Agents. Int. J. Mol. Sci..

[40] López-Valverde N., López-Valverde A., Montero J., Rodríguez C., Macedo de Sousa B., Aragoneses J.M. (2023). Antioxidant, anti-inflammatory and antimicrobial activity of natural products in periodontal disease: A comprehensive review. Front. Bioeng. Biotechnol..

