# Supplementary material for: Ameliorative Effect of Ginsenoside Rg6 in Periodontal Tissue Inflammation and Recovering Damaged Alveolar Bone
Source: Molecules. 2023 Dec 20;29(1):46. doi: 10.3390/molecules29010046 (PMC10779481; doi:10.3390/molecules29010046)
Supplement: Supplementary file 1 [file molecules-29-00046-s001.zip › molecules-2700680-supplementary.pdf]

Supplementary File

Ameliorative Effect of Ginsenoside Rg6 in Periodontal Tissue Inflammation and Recovering Damaged Alveolar Bone

Won-Jin Lee [1,†], Eun-Nam Kim [1,†], Nguyen Minh Trang [1], Jee-Hyun Lee [2], Soo-Hyun Cho [2], Hui-Ji Choi [1], Gyu-Yong Song [1,2,*] and Gil-Saeng Jeong [1,*]

[1] College of Pharmacy, Chungnam National University, Daejeon 34134, Republic of Korea; wonjin2373@gmail.com (W.-J.L.); enkim@cnu.ac.kr (E.-N.K.); ngminhtrang52@gmail.com (N.M.T.); gmlwl5@naver.com (H.-J.C.)
[2] AREZ Co. Ltd., Daejeon 34036, Republic of Korea; i12ulove@arez365.com (J.-H.L.); sweetylover@arez365.com (S.-H.C.)
* Correspondence: gysong@cnu.ac.kr (G.-Y.S.); gsjeong@cnu.ac.kr (G.-S.J.); Tel.: +82-42-821-5926 (G.-Y.S.); +82-42-821-5937 (G.-S.J.)
† These authors contributed equally to this work.

List of Figures

Figure S1. Mass spectrometry of G-Rg6

Figure S2. Nuclear Magnetic Resonance (NMR) spectrum of G-Rg6

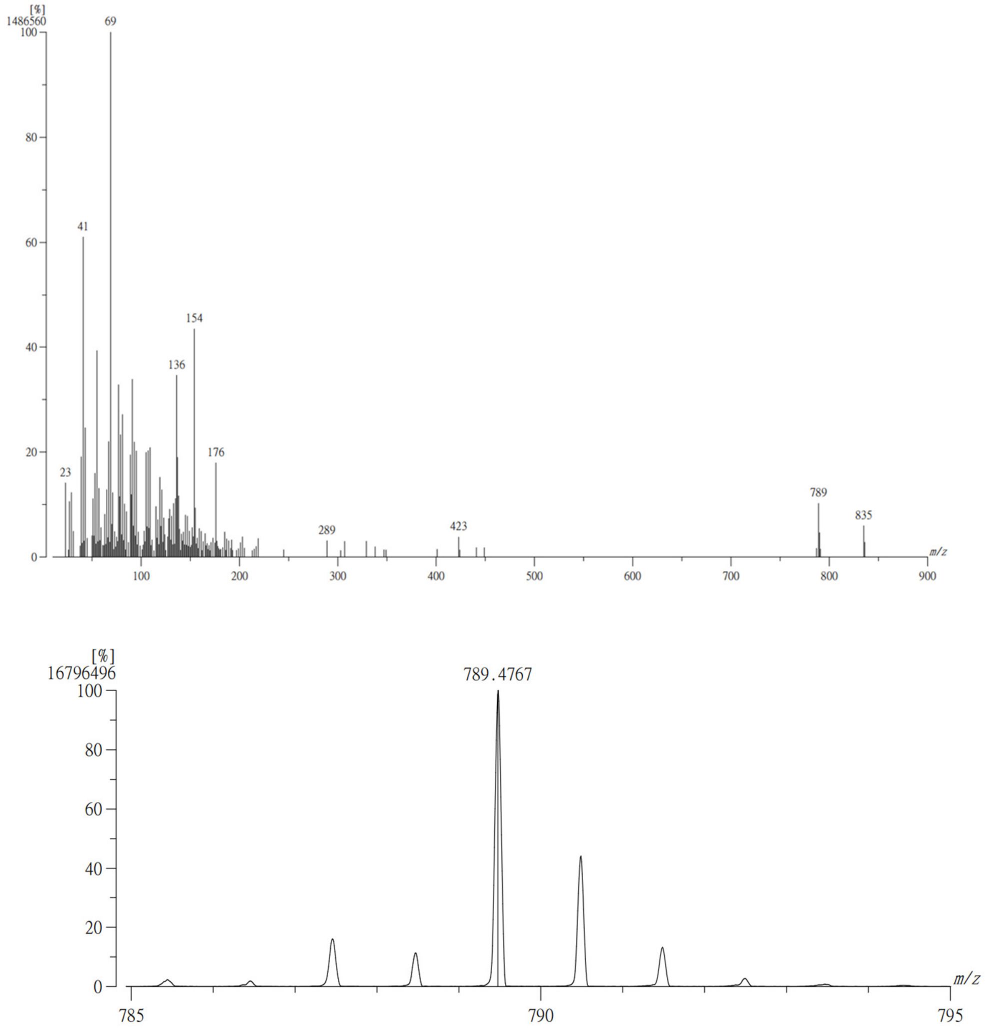

	Observed m/z	Int%	Err[ppm / mmu]	U.S.	Composition
1	789.4767	100.00	-2.8 / -2.2	10.5	C44 H69 O12
2			+0.3 / +0.2	7.5	C42 H70 O12 Na

Figure S1. Mass spectrometry of G-Rg6.

^{13}C-NMR

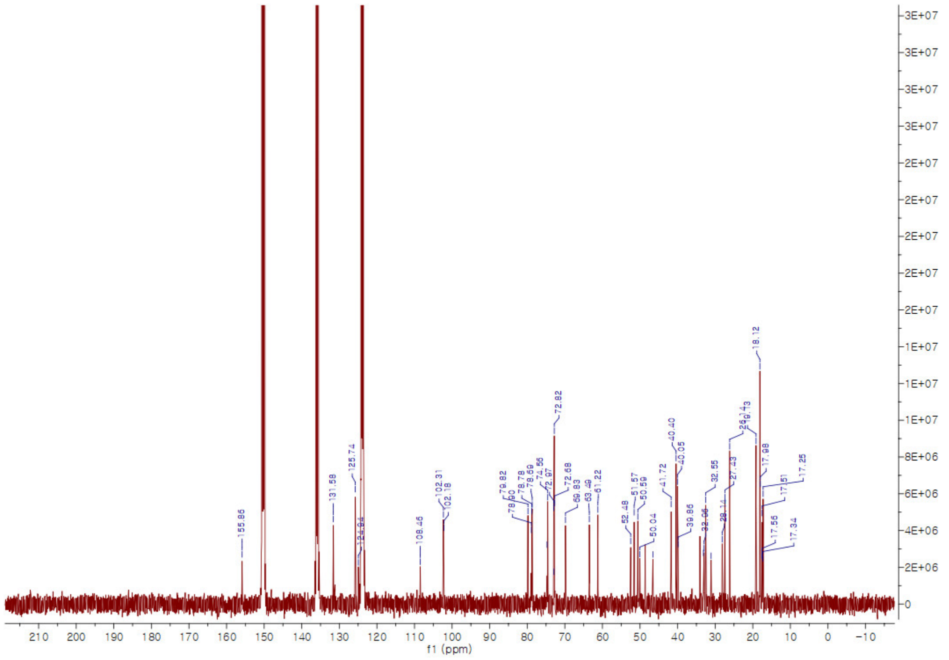

^{1}H-NMR

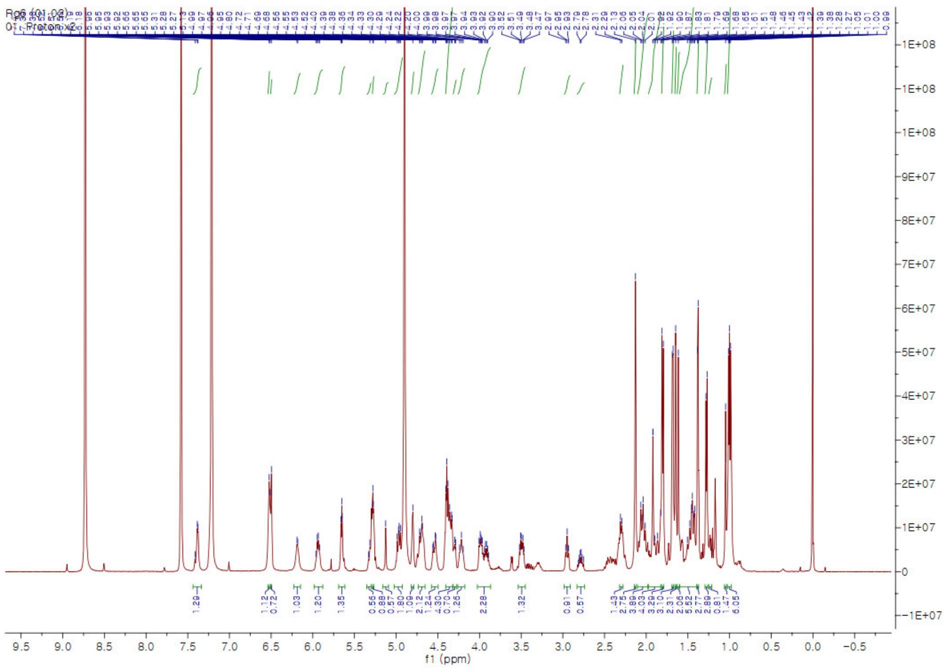

Figure S2. Nuclear Magnetic Resonance (NMR) spectrum of G-Rg6.
